# 2021. Efficacy of Chronic Antimicrobial Suppressive Therapy in Ventricular Assist Device Infections

**DOI:** 10.1093/ofid/ofac492.1645

**Published:** 2022-12-15

**Authors:** Nicholas S Teran, Hannah Russo, M Rizwan Sohail, Kady Phe

**Affiliations:** Baylor St. Luke's Medical Center, Houston, Texas; Baylor St. Luke's Medical Center, Houston, Texas; Baylor College of Medicine, Houston, Texas; Baylor St. Luke's Medical Center, Houston, Texas

## Abstract

**Background:**

Ventricular assist devices (VAD) prolong life expectancy or serve as a bridge to heart transplant in end stage heart failure but are not without risks. These patients are at high risk for device-related infections with a propensity for relapse. Indications and efficacy of chronic antimicrobial suppression (CAS) to prevent relapse are not well defined.

**Methods:**

We conducted a retrospective study of all adult patients with VAD infections at our institution between 1/1/2013 and 10/31/2020. VAD-specific or VAD-related infections were defined by the International Society for Heart and Lung Transplantation criteria. Patients were stratified by receipt of CAS or no CAS after completing index infection treatment. Relapsed infections were defined as those recurred at the same site caused by the same organism seen in the index infection. Time-to-relapse infection was defined as the time in days between the index infection and first relapse. The primary outcome was CAS efficacy measured by time-to-relapse in VAD-specific or -related infections.

**Results:**

A total of 83 patients were included with a documented VAD-specific (n = 68) or VAD-related (n = 15) infection. Driveline (n = 66) and bloodstream (n = 13) infections were the most common VAD-specific and VAD-related infections, respectively. For the index infection, all patients received antimicrobial treatment and 16.4% required incision and drainage. Staphylococcus aureus was most frequently isolated in 46 cases. Oral minocycline was the most commonly utilized agent in 22 of 47 patients receiving CAS. Agent-specific CAS efficacy was unable to be assessed due to the small sample. In patients who received CAS, 57% experienced a relapse infection compared to 81% in patients not receiving CAS (p = 0.03). The median time to relapse was 151 days (IQR 78-247) and 57 days (IQR 36-114) in the CAS and non-CAS groups, respectively (p < 0.01).

**Conclusion:**

The incidence of relapse infection in the setting of CAS was lower compared to those not receiving CAS. Patients receiving CAS were relapse-free for a significantly longer duration. Larger studies are warranted to assess if one agent confers a benefit over another.

**Disclosures:**

**M. Rizwan Sohail, MD**, Aziyo Biologics: Honoraria|Boston Scientific Corporation: Honoraria|Medtronic: Grant/Research Support|TRYX: Grant/Research Support.

